# The Effect of Mitochondrial Supplements on Mitochondrial Activity in Children with Autism Spectrum Disorder

**DOI:** 10.3390/jcm6020018

**Published:** 2017-02-13

**Authors:** Leanna M. Delhey, Ekim Nur Kilinc, Li Yin, John C. Slattery, Marie L. Tippett, Shannon Rose, Sirish C. Bennuri, Stephen G. Kahler, Shirish Damle, Agustin Legido, Michael J. Goldenthal, Richard E. Frye

**Affiliations:** 1 Arkansas Children’s Research Institute, Little Rock, AR 72202, USA; lmdelhey@uams.edu (L.M.D.); ekimkilinc@gmail.com (E.N.K.); jcslattery@uams.edu (J.C.S.); mltippett@uams.edu (M.L.T.); srose@uams.edu (S.R.); scbennutri@uams.edu (S.C.B.); kahlerstepheng@uams.edu (S.G.K.); 2 Department of Pediatrics, University of Arkansas for Medical Sciences, Little Rock, AR 72202, USA; 3 Child and Adolescent Department, Mental Health Centre, West China Hospital of Sichuan University, Chengdu 610041, China; dr.yinli@hotmail.com; 4 Department of Pediatrics, Drexel University College of Medicine, Neurology Section, St. Christopher’s Hospital for Children, Philadelphia, PA 19134, USA; shirish.damle@drexelmed.edu (S.D.); agustin.legido@drexelmed.edu (A.L.); michael.goldenthal@drexelmed.edu (M.J.G.)

**Keywords:** antioxidants, autism spectrum disorder, B12, Complex I, Complex IV, electron transport chain, fatty acids, folate, mitochondrial disease, mitochondrial dysfunction

## Abstract

Treatment for mitochondrial dysfunction is typically guided by expert opinion with a paucity of empirical evidence of the effect of treatment on mitochondrial activity. We examined citrate synthase and Complex I and IV activities using a validated buccal swab method in 127 children with autism spectrum disorder with and without mitochondrial disease, a portion of which were on common mitochondrial supplements. Mixed-model linear regression determined whether specific supplements altered the absolute mitochondrial activity as well as the relationship between the activities of mitochondrial components. Complex I activity was increased by fatty acid and folate supplementation, but folate only effected those with mitochondrial disease. Citrate synthase activity was increased by antioxidant supplementation but only for the mitochondrial disease subgroup. The relationship between Complex I and IV was modulated by folate while the relationship between Complex I and Citrate Synthase was modulated by both folate and B12. This study provides empirical support for common mitochondrial treatments and demonstrates that the relationship between activities of mitochondrial components might be a marker to follow in addition to absolute activities. Measurements of mitochondrial activity that can be practically repeated over time may be very useful to monitor the biochemical effects of treatments.

## 1. Introduction

Primary mitochondrial disease, as well as secondary mitochondrial dysfunction, is becoming increasingly recognized [1]. Indeed, the contribution of the mitochondria to many diverse, common disorders such as diabetes, obesity, cancer and heart, neurologic and psychiatric disease is significant. What is less well known is the optimal treatment for mitochondrial disease and dysfunction. Several expert opinion papers provide insight into the recognized management of patients with mitochondrial disease. However, such expert opinion is based on a paucity of clinical evidence [2,3]. Although new novel therapies have undergone increasing investigation recently, most of the published information remains in the preclinical stage, isolated to evidence from model organisms [4]. Although clinical trials have been conducted, the rarity of certain mitochondrial diseases; small subject numbers; heterogeneity in symptoms; severity of specific mitochondrial diseases; short treatment and follow-up periods; variability in outcomes measures; and the use of measures that are not specifically designed to measure mitochondrial outcomes, are all factors which probably contribute to the lack of positive findings in clinical trials [5,6]. Thus, there has been a recent call to develop new biomarkers of mitochondrial function that can be used in future well-designed clinical trials [7].

Biochemical measurements of mitochondrial function can be variable or difficult to obtain. For example, laboratory measures are commonly very sensitive to collection techniques and laboratory processing, resulting in significant variability. Magnetic resonance spectroscopy is a promising technique to non-invasively measure energy metabolism in muscle and brain tissues, but is limited to centers with specialized equipment. In addition, to date, none of these markers have been found to be systematically altered in high-quality clinical trials [7]. Direct measurement of mitochondrial function by enzymology typically requires biopsies that are somewhat invasive, limiting their ability to be repeated to follow the disease status. In 2012, Goldenthal et al. developed and validated the non-invasive buccal swab technique, demonstrating the correspondence between enzymology measurements in buccal tissue and muscle biopsy in individuals with mitochondrial disease [8]. The buccal swab technique has been used to measure mitochondrial function in individuals with mitochondrial disease [8,9,10], specific genetic syndromes [10,11] and Autism Spectrum Disorder (ASD) [12,13].

ASD is a behaviorally defined disorder which now affects ~2% of children [14]. Recent studies suggest that ASD is linked to mitochondrial dysfunction [13,15,16], although the exact nature of mitochondrial abnormalities in ASD appears to be complicated. For example, classic mitochondrial disease is found in 5% of children with ASD [16], yet up to 50% of children with ASD may have biomarkers of mitochondrial dysfunction [16,17] and a higher rate of abnormal electron transport chain (ETC) activity is found in immune cells [18,19] and post-mortem brain tissue [20]. Perhaps more unique is the fact that ETC activity in muscle [21,22], skin [23], buccal cells [11,12,13] and the brain [20] has been documented to be significantly increased, rather than decreased, in individuals with ASD, consistent with in vitro data showing elevated mitochondrial respiration in cell lines derived from children with ASD [24,25]. More recently, mitochondrial respiration in cell lines has been shown to be related to the stereotyped behaviors and restricted interests subscale on the Autism Diagnostic Observation Scale (ADOS) with elevated respiratory rates corresponding to worse behavior [26].

Individuals with ASD are a particularly important group of patients that would benefit from a biomarker of mitochondrial dysfunction as well as a marker of the effect of treatments on mitochondrial function. First, the great majority of children with ASD do not have genetic mutations to explain their mitochondrial dysfunction [16], making diagnosis complicated. Second, many children with ASD are treated with supplements that potentially target the mitochondrial but it is unclear whether such treatments influence mitochondrial function [27]. Understanding which treatments would be most helpful and effective for children with ASD, especially on an individual basis, would be tremendously helpful for guiding treatment in a personalized medicine fashion.

In this study, we aimed to ask whether the functional effect of common treatments that target the mitochondria can be measured with a non-invasive buccal swab technique and what are the measures that might be sensitive to the effect of treatment. To this end, we measured the activity of ETC Complex I and IV as well as Citrate Synthase. We not only examined the absolute level of activity of mitochondrial components, but also the relationship between the components, to better understand whether treatments not only modulated the activity level but how the mitochondrial components work together. To this end, we utilized the data from our study of the natural history of mitochondrial function in children with ASD to examine the mitochondrial function on individuals taking and abstaining from common treatments that affect the mitochondrial. Since specific supplements were not systematically manipulated, it is not possible to equate the findings from this study to a clinical trial of specific supplements. Rather, this study is designed to answer the question of whether the technique and measurements used in the study show promise for future research.

## 2. Material and Methods

The study was approved by the Institutional Review Board at the University of Arkansas for Medical Sciences (Little Rock, AR, USA) under two protocols (#137162 originally approved on August 7th 2012 and #136272 originally approved on May 25th 2012). Parents of participants provided written informed consent.

### 2.1. Participants 

#### 2.1.1. Autism Spectrum Disorder

Individuals with ASD who met the inclusion and exclusion criteria had mitochondrial function measured up to four times using the buccal swab technique described below. Inclusion criteria included: (i) age 3 to 14 years of age and (ii) ASD diagnosis. Exclusion criteria included prematurity.

The ASD diagnosis was defined by one of the following: (i) a gold-standard diagnostic instrument such as the ADOS and/or Autism Diagnostic Interview-Revised; (ii) the state of Arkansas diagnostic standard, defined as the agreement of a physician, psychologist and speech therapist; and/or (iii) Diagnostic Statistical Manual (DSM) diagnosis by a physician along with standardized validated questionnaires and diagnosis confirmation by the Principal Investigator.

#### 2.1.2. Mitochondrial Disease

Individuals included in this study were screened for mitochondrial disease through a standard clinical protocol [23,28]. Mitochondrial disease was diagnosed in a portion of the individuals using a combination of biochemical, enzymology and genetic testing. In general, the modified Walkers criterion was used to diagnose mitochondrial disease, although in some cases with clear repeated biochemical abnormalities with clinical symptomatology that lacked an identifiable genetic component, the Morava criterion was used [15].

#### 2.1.3. Historical Healthy Controls

Controls of similar age and gender included 68 healthy individuals without neurological disease as described in previous studies [12]. Controls ranged in age from 3 to 21 years of age [mean (Standard Deviation (SD)) 10.1 years (4.6 years)] with 33 (49%) being female. In a previous report, it was found that there was no correlation between enzyme activities and age and no difference in protein activities across ethnicity or race in both controls and mitochondrial disease patients [8].

### 2.2. Measures of Mitochondrial Function

The buccal cells were collected using Catch-All Buccal Collection Swabs (Epicentre Biotechnologies, Madison, WI, USA). Four swabs were collected by firmly pressing a swab against the inner cheek while twirling for 30 s. Swabs were clipped and placed in 1.5 mL microcentrifuge tubes that were labeled and placed on dry ice for overnight transportation to the Goldenthal laboratory.

Buccal extracts were prepared using an ice-cold buffered solution (Buffer A, ABCAM, Cambridge, MA, USA) containing protease inhibitor cocktail and membrane solubilizing non-ionic detergent and cleared of insoluble cellular material by high speed centrifugation at 4 °C. Duplicate aliquots of the protein extract were analyzed for protein concentration using the bicinchoninic acid method (Pierce Biotechnology, Rockford, IL, USA). Samples were typically stored at −80 °C for up to 1 week prior to enzymatic analysis.

Dipstick immunocapture assays measured ETC Complex I activity using 50 µg extracted protein [8,9,10]. Signals were quantified using a Hamamatsu immunochromato MS 1000 Dipstick reader (ABCAM, Cambridge, MA, USA). Raw mABS (milliAbsorbance) results were corrected for protein concentration and data were expressed as percentages of the values obtained with control extracts run on the same assay. ETC Complex IV and Citrate Synthase (CS) activity was assessed using standard spectrophotometric procedures in 0.5 mL reaction volume. Specific activities of respiratory complexes and citrate synthase were initially expressed as nanomoles/min/mg protein. This activity was then normalized to control values so that the final value represented a z-score. This allowed for the direct comparison of activities across complexes and citrate synthase.

### 2.3. Statistical Analysis

Analyses were performed using SAS 9.4 (SAS Institute Inc., Cary, NC, USA). Graphs were produced using Excel version 14.0 (Microsoft Corp, Redmond, WA, USA). Normal control values for mitochondrial function were based upon the established controls from the Goldenthal laboratory [12]. A mixed-model linear regression was used to account for both within-subject variation from repeated measurements on the same individual as well as between-subject variation such as mitochondrial disease subgroup. The module “glimmix” in SAS was used with an *p* ≤ 0.05. 

A series of analyses first examined the effect of specific supplements on overall normalized mitochondrial activities including an interaction the with mitochondrial disease subgroup (mitochondrial disease vs no mitochondrial disease). Main effects and interactions in the model are F-distributed so they were evaluated using a F-test. If the interaction was significant, post-hoc orthogonal contrasts were used to determine whether the effect of the supplement was specific to one subgroup. Post-hoc orthogonal contrasts are t-distributed and thus were evaluated using a t-distribution. The supplements that were found to have a significant effect were then entered into a stepwise backward mixed-model regression (with mitochondrial disease subgroup interaction if significant in individuals regressions) with a criteria of *p* ≤ 0.05 to keep in the model. Essentially, at each step, the variable with the highest *p*-value was eliminated and the model was recalculated until all of the variables in the model were significant at the *p* ≤ 0.05 level. Of course, variables that were dependents of an interaction were kept in the model irrespective of their significance.

Similarly, a series of analyses examined the effect of specific supplements on the relationship between the normalized mitochondrial component activities, including an interaction with the mitochondrial disease subgroup. If the interaction was significant, post-hoc orthogonal contrasts were used to determine whether the effect of the supplement on the relationship between the mitochondrial components (i.e., the slope of the regression) was specific to one subgroup. The supplements that were found to be significant were then entered into a stepwise backward mixed-model regression (with mitochondrial disease subgroup interaction if significant in the single supplement models). As before, at each step, the variable with the highest *p*-value was eliminated and the model was recalculated until all of the variables in the model were significant at the *p* ≤ 0.05 level. Variables that were dependents of an interaction were kept in the model irrespective of their significance.

## 3. Results

### 3.1. Participants

A total of 127 individuals with ASD who met the inclusion and exclusion criteria had mitochondrial function measured. Of the 127 participants, 38 had mitochondrial function measured twice, seven had mitochondrial function measured three times and one participant had mitochondrial function measured four times. The mean age at the first mitochondrial function measurement was 8.3 years (SD = 4.0 years) with 77% being male. Age and gender were entered into the regressions initially but were found not to be significant so they were not included in the subsequent analyses. A total of 15% of the sample was clinically diagnosed with mitochondrial disease. Mitochondrial disease was found to be a significant factor in the regression analyses so it was included in most analyses. The only exceptions were for the analysis of multivitamin (MVI) and herbal supplements where there were too few individuals with mitochondrial disease taking these supplements for a valid analysis of this effect by subgroup.

### 3.2. Supplements

Participants were not on any supplements for 118 measurements. Of those measurements, 26 participants were naïve to supplements, whereas for 92 measurements, supplements were held for an average of 21.3 days (SD 15.4 days; Range 1–61 days) before the measurements. For 55 measurements, supplements were given as scheduled. The specific supplements taken by the participants are outlined in Table 1. The factors of (a) time since taking the supplement when supplements were held, and (b) naivety to supplementation, were entered into the regressions initially but were found not to be significant so they were not included in the subsequent analyses.

### 3.3. Supplement Effect on Mitochondrial Complexes and Citrate Synthase Activity

#### 3.3.1. Normalized Complex I Activity

Carnitine, antioxidant and other vitamin supplementation were associated with significantly higher Complex I activity [F(1.44) = 7.58, *p* < 0.01, F(1.44) = 6.54, *p* = 0.01, F(1.44) = 6.70, *p* = 0.01, respectively] (See Table 2).

Fatty acids and folate supplementation influenced Complex I activity with this influence different for the mitochondrial disease subgroup (See Table 3). Fatty acid supplementation significantly increased Complex I activity overall [F(1.44) = 16.86, *p* < 0.0005] but also interacted with mitochondrial disease subgroup [F(1.44) = 4.53, *p* < 0.05]. This interaction resulted from this increase being more marked for the mitochondrial disease subgroup when the subgroups were analyzed separately despite the fact that both the no mitochondrial disease [t(44) = 2.05, *p* < 0.05] and the mitochondrial disease [t(44) = 3.56, *p* < 0.001] subgroups demonstrated a significant effect of supplementation. Folate supplementation significantly increased Complex I activity overall [F(1.44) = 11.15, *p* < 0.005] but there was an interaction with mitochondrial disease subgroup [F(1.44) = 5.61, *p* < 0.05]. This interaction resulted from folate only significantly influencing Complex I activity in the mitochondrial disease group [t(44) = 3.28, *p* < 0.005] when the subgroups were analyzed separately.

Complex I was not significantly influenced by amino acid, B12, MVI, B vitamins, Coenzyme Q10 (CoQ10), or herbal supplementation.

To determine which supplements were driving the effect on Complex I activity, the supplements that demonstrated a significant effect on Complex I activity were entered into a stepwise backwards regression. The regression demonstrated that fatty acids supplementation significantly increased Complex I activity [F(1.43) = 6.39, *p* < 0.05] without an interaction between subgroups and that the effect of folate on Complex I activity was influenced by the subgroup [F(1.43) = 5.94, *p* < 0.05] since the effect of folate was isolated to the mitochondrial disease group [t(43) = 2.42, *p* < 0.05].

#### 3.3.2. Normalized Citrate Synthase Activity

Fatty acids, folate and antioxidant supplementation influenced Citrate Synthase activity with this influence being different for the mitochondrial disease subgroup (See Table 4). Fatty acid supplementation significantly increased Citrate Synthase activity [F(1.44) = 9.58, *p* < 0.005] but also interacted with the mitochondrial disease subgroup [F(1.44) = 4.69, *p* < 0.05]. This interaction resulted from fatty acids significantly influencing Citrate Synthase activity only in the mitochondrial disease group [t(44) = 3,02, *p* < 0.005] when the subgroups were analyzed separately. Folate supplementation significantly increased Citrate Synthase activity [F(1.44) = 7.00, *p* = 0.01] but also interacted with the mitochondrial disease subgroup [F(1.44) = 7.56, *p* < 0.01]. This interaction resulted from folate significantly influencing Citrate Synthase activity only in the mitochondrial disease group [t(44) = 3.13, *p* < 0.005] when the subgroups were analyzed separately. Antioxidant supplementation significantly increased Citrate Synthase activity [F(1.44) = 7.37, *p* < 0.01] but also interacted with the mitochondrial disease subgroup [F(1.44) = 8.30, *p* < 0.01]. This interaction resulted from antioxidants significantly influencing Citrate Synthase activity only in the mitochondrial disease group [t(44) = 3.22, *p* = 0.01] when the subgroups were analyzed separately.

To determine which supplements were driving the effect on Complex I activity, the supplements that demonstrated a significant effect on Citrate Synthase activity were entered into a stepwise backwards elimination regression. The regression only selected antioxidant supplementation for improvement in Citrate Synthase activity (results same as above).

Citrate Synthase was not significantly influenced by amino acid, B12, B vitamins, multivitamin, CoQ10, carnitine, other vitamins or herbal supplementation.

#### 3.3.3. Normalized Complex IV Activity

Normalized Complex IV activity was not significantly influenced by amino acid, B12, B vitamins, CoQ10, carnitine, other vitamins, herbal, fatty acids, folate, multivitamin or antioxidant supplementation.

### 3.4. Supplement Effect on Relationship between Mitochondrial Complexes and Citrate Synthase Activity

#### 3.4.1. The Relationship between Normalized Complex I and Complex IV Activity

The relationship between Normalized Complex I and Complex IV activity was not influenced by amino acids, multivitamin, B12, herbal, other vitamins or CoQ10. B vitamins, fatty acids, folate, antioxidants and carnitine all influenced the relationship between Normalized Complex I and Complex IV activity with this relationship influenced by whether or not the participant was in the mitochondrial disease subgroup.

Most of the supplements predominantly influenced the mitochondrial disease subgroup. B vitamins significantly influenced the relationship between complexes [F(1.40) = 5.88, *p* < 0.05] with this effect interacting with the mitochondrial disease subgroup [F(1.40) = 7.45, *p* < 0.01] since the effect was significant only in the mitochondrial disease subgroup [t(40) = 3.09, *p* < 0.005]. Fatty acids significantly influenced the relationship between complexes [F(1.40) = 12.64, *p* = 0.001] with this effect interacting with the mitochondrial disease subgroup [F(1.40) = 7.45, *p* < 0.01] since the effect was significant only in the mitochondrial disease subgroup [t(40) = 3.17, *p* < 0.005]. Antioxidants significantly influenced the relationship between complexes [F(1.40) = 14.34, *p* = 0.0005] with this effect interacting with the mitochondrial disease subgroup [F(1.40) = 10.01, *p* < 0.005] because the effect was only significant in the mitochondrial disease subgroup [t(40) = 3.83, *p* < 0.0005]. Carnitine significantly influenced the relationship between complexes [F(1.40) = 7.64, *p* < 0.01] with this effect interacting with the mitochondrial disease subgroup [F(1.40) = 4.49, *p* < 0.05] because the effect was only significant in the mitochondrial disease subgroup [t(40) = 2.71, *p* < 0.01].

Folate influenced the relationship between Normalized Complex I and Complex IV activity [F(1.40) = 18.13, *p* = 0.0001] with the effect of folate being influenced by the mitochondrial disease subgroup [F(1.40) = 7.04, *p* = 0.01]. The effect of folate was more marked in the mitochondrial disease subgroup [t(40) = 3.75, *p* < 0.001] than the non-mitochondrial disease subgroup [t(40) = 2.07, *p* < 0.05] but folate did influence mitochondrial function for both those with and without mitochondrial disease.

**Figure 1:**
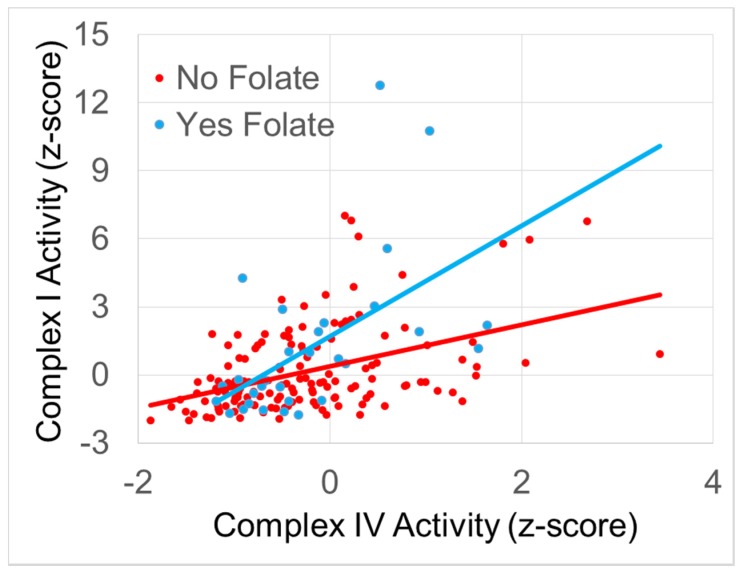
The relationship between Normalized Complex I and IV activity. Folate supplementation is associated with a significantly greater slope in the relationship between complex activities.

To determine which supplements were driving the effect, the supplements that demonstrated significant effects were entered into a stepwise backwards regression. The regression demonstrated that folate supplementation significantly improved the relationship between Normalized Complex I and Complex IV activity. Figure 1 depicts the effect of folate supplementation on the relationship between Normalized Complex I and Complex IV activity. Those on folate supplementation had a stronger relationship between Complex I and Complex IV activity as compared to individuals not on folate supplementation. The regression coefficients suggest that each increase in Complex IV activity results in a 2.4 times increase in Complex I activity if an individual was on folate supplementation while this increase was only 0.9 times if an individual was not on folate supplementation. 

#### 3.4.2. The Relationship between Normalized Complex I and Citrate Synthase Activity

The relationship between Normalized Complex I and Citrate Synthase activity was not influenced by amino acids, MVI, fatty acids, herbal, other vitamins or CoQ10. B vitamins, B12, folate, antioxidants and carnitine all influenced the relationship between Normalized Complex I and Complex IV activity. 

Some supplements predominately influenced the mitochondrial disease subgroup. The effect of B vitamins on the relationship between mitochondrial components was significantly influenced by the mitochondrial disease subgroup [F(1.40) = 5.44, *p* < 0.05] because the effect was isolated to the mitochondrial disease subgroup [t(40) = 2.43, *p* < 0.05]. B12 significantly influenced the relationship between mitochondrial components [F(1.40) = 4.27, *p* < 0.05] with this effect being influenced by the mitochondrial disease subgroup [F(1.40) = 6.24, *p* = 0.01] because the effect was only significant for the mitochondrial disease subgroup [t(40) = 2.53, *p* < 0.05].

Folate, antioxidants and carnitine appear to influence the relationship between Normalized Complex I and Citrate Synthase activity without a difference in this effect across subgroups [F(1.40) = 21.36, *p* < 0.0001, F(1.40) = 7.09, *p* = 0.01 and F(1.40) = 4.74, *p* < 0.05, respectively].

**Figure 2:**
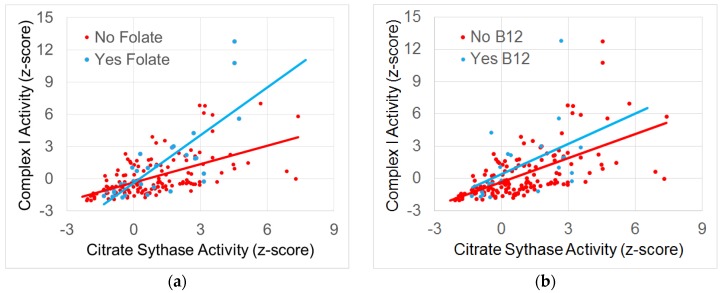
The relationship between normalized Complex I and Citrate Synthase activity. (**a**) Folate and (**b**) B12 supplementation are associated with a significantly greater slope in the relationship between Complex I and Citrate Synthase.

To determine which supplementation was driving the effect, the supplements that demonstrated significant effects were entered into a stepwise backwards elimination regression. The regression demonstrated that folate and B12 supplementation significantly altered the relationship between Normalized Complex I and Citrate Synthase activity [F(1.41) = 28.23, *p* < 0.0001 and F(1.41) = 8.35, *p* < 0.005, respectively] without an interaction between subgroups. Figure 2 depicts these relationships. Folate increased the slope of the relationship between Normalized Complex I and Citrate Synthase activity such that any increase in Citrate Synthase resulted in a 1.5 times increase in Normalized Complex I activity if an individual was on folate, whereas it resulted in only a 0.5 times increase in Normalized Complex I activity if the individual was not on folate. For B12 supplementation, an increase in Citrate Synthase resulted in a 0.8 times increase in Complex I activity if an individual was supplementing with B12, whereas this was only 0.6 times if an individual was not supplementing with B12.

#### 3.4.3. The Relationship between Normalized Complex IV and Citrate Synthase Activity

None of the supplements were found to influence the relationship between Normalized Complex IV and Citrate Synthase activity.

## 4. Discussion

This study examined the effect of common mitochondrial treatments on specific mitochondrial components in a group of children diagnosed with ASD, some of which also were diagnosed with co-morbid mitochondrial disease. Measurement of mitochondrial function is important in ASD since many children with ASD appear to have mitochondrial dysfunction even if they are not diagnosed with classic mitochondrial disease. Furthermore, the influence of mitochondrial treatment in ASD is important as randomized controlled clinical trials have demonstrated that common treatments for mitochondrial disease, such as L-carnitine, improve ASD symptoms, suggesting that such treatments may have a role in the treatment of ASD [27]. However, what remains unclear is whether these treatments are targeting mitochondrial function per se.

In addition, in this study, we examined not only whether common mitochondrial supplements affect the absolute levels of activity of three mitochondrial components, but whether the treatments alter the relationship between the components. This may be important, as optimal coupling of the various mitochondrial components is essential for the mitochondria to function optimally.

Results from this study suggested that several common mitochondrial supplements such as fatty acids and antioxidants appeared to influence Complex I and Citrate Synthase activity, respectively, with this influence being more marked for the mitochondrial disease subgroup. This is not unexpected as such treatments are sometimes recommended for individuals with mitochondrial disease, particularly antioxidants. Fatty acids are not always recommended for individuals with mitochondrial disease. Several studies have suggested that omega 3 fatty acids, which are the most fatty acids prescribed to children with ASD, have positive effects on behavior [29]. Interestingly, recent research has highlighted the role of fatty acids in preserving mitochondrial function in such diseases as stroke [30] and cancer [31] as well as improving muscle health through modulation of mitochondrial function [32]. The findings that these effects were more marked in the mitochondrial disease subgroup suggest that these treatments are indeed targeting and improving mitochondrial function and further suggest that certain treatments may be best targeted to subpopulations of individuals with ASD.

Folate was found to be potentially important in modulating the relationship between both Complex I and IV and Complex I and Citrate Synthase, while B12 appeared to be potentially important in the relationship between Complex I and Citrate Synthase. In a clinical trial on individuals with ASD, the combination of B12 and folate has been shown to improve cognitive development [28] and glutathione [33], the major intrinsic antioxidant that is essential for protecting the mitochondrial. In another clinical trial, B12 alone has been shown to improve methylation in individuals with ASD [34]. Folate is also essential for mitochondrial function as one-carbon metabolism is highly compartmentalized [35]. Most notable in the context of mitochondrial disease, is that mitochondria often replicate to compensate for poorly functioning mitochondria. Since mitochondria contain their own DNA, folate is needed for the synthesis of purines and pyrimidine nucleotides [35]. Thus, given the important role of folate in many critical cellular processes, it should not be surprising that it was found to be important in mitochondrial function.

This study has many limitations, including the lack of systematically manipulating the treatment studied and simultaneous treatments with multiple supplements in many cases. In addition, the subgroups of individuals with mitochondrial disease were not diagnosed with one specific mitochondrial disease. Nevertheless, this study provides a novel framework to build upon in order to consider the development of alternative methods for monitoring mitochondrial function. 

## 5. Conclusions

This study provides empirical support for common mitochondrial treatments and demonstrates that the relationship between activities of mitochondrial components might be a marker to follow in addition to absolute activities. In addition, measurements of mitochondrial activity that can be practically repeated over time, especially those that are non-invasive such as the buccal swab technique, may be very useful to monitor the biochemical effects of mitochondrial targeted treatments.

## Figures and Tables

**Table 1:** Supplements taken by participants.

Supplement	% Taking Regularly	% Taking During Mitochondrial Testing	% Holding During Mitochondrial Testing
Amino Acids	23%	4%	19%
B12	38%	13%	25%
B Vitamins	36%	10%	26%
Carnitine	42%	13%	29%
Coenzyme Q10	36%	9%	27%
Fatty Acids	45%	16%	29%
Folate	54%	16%	38%
Herbal	17%	6%	11%
Multivitamin	26%	12%	14%
Antioxidants	46%	14%	32%
Other Vitamins	49%	14%	35%

**Table 2:** Means (Standard Error) of Normalized Complex I activity on and off supplements.

Supplement	Off Supplement	On Supplement
Carnitine	−0.1 (0.28)	1.4 (0.46)
Antioxidants	0.1 (0.26)	1.5 (0.50)
Other Vitamins	0.1 (0.27)	1.4 (0.45)

**Table 3:** Means (Standard Error) of Normalized Complex I activity on and off supplements by Mitochondrial Disease group. Supplements that are confirmed to be significant in the stepwise regression are bolded and italicized.

Supplement	No Mitochondrial Disease		Mitochondrial Disease	
	Off Supplement	On Supplement	Off Supplement	On Supplement
***Fatty Acids***	***0.1 (0.20)***	***1.2 (0.48)***	***−0.3 (0.47)***	***3.1 (0.84)***
Folate	0.2 (0.21)	0.7 (0.5)	***−0.3 (0.49)***	***2.7 (0.79)***

**Table 4:** Means (Standard Error) of Normalized Citrate Synthase activity on and off supplements by Mitochondrial Disease group. Supplements that are confirmed to be significant in the stepwise regression are bolded and italicized.

Supplement	No Mitochondrial Disease		Mitochondrial Disease	
	Off Supplement	On Supplement	Off Supplement	On Supplement
Fatty Acids	0.8 (0.17)	1.2 (0.40)	0.3 (0.40)	2.6 (0.70)
Folate	0.9 (0.18)	0.8 (0.40)	0.2 (0.42)	2.4 (0.66)
***Antioxidants***	0.9 (0.17)	0.8 (0.42)	***0.2 (0.41)***	***2.7 (0.71)***
